# Oligodendrocyte-specific ATF4 inactivation does not influence the development of EAE

**DOI:** 10.1186/s12974-019-1415-6

**Published:** 2019-02-01

**Authors:** Yuan Yue, Milos Stanojlovic, Yifeng Lin, Gerard Karsenty, Wensheng Lin

**Affiliations:** 1 Department of Neuroscience, University of Minnesota, Minneapolis, MN 55455 USA; 2 Institute for Translational Neuroscience, University of Minnesota, Minneapolis, MN 55455 USA; 3 Department of Genetics and Development, Columbia University Medical Center, New York, NY 10032 USA; 4 Key Laboratory of Birth Defects, Children’s Hospital of Fudan University, Shanghai, 201102 China

**Keywords:** Multiple sclerosis, EAE, PERK, ATF4, Neurodegeneration, Oligodendrocyte

## Abstract

**Background:**

Multiple sclerosis (MS) and its animal model, experimental autoimmune encephalomyelitis (EAE), are inflammatory demyelinating and neurodegenerative diseases of the CNS. Although recent studies suggest the neuroprotective effects of oligodendrocytes in neurodegenerative diseases, it remains unknown whether oligodendrocyte death induced by inflammatory attacks contributes to neurodegeneration in MS and EAE. Upon endoplasmic reticulum (ER) stress, activation of pancreatic ER kinase (PERK) promotes cell survival through induction of activating transcription factor 4 (ATF4) by phosphorylating eukaryotic translation initiation factor 2α (eIF2α). We have generated a mouse model that allows for temporally controlled activation of PERK specifically in oligodendrocytes. Our previous study has demonstrated that PERK activation specifically in oligodendrocytes attenuates EAE disease severity and ameliorates EAE-induced oligodendrocyte apoptosis, demyelination, and axon degeneration, without altering inflammation.

**Methods:**

We determined whether oligodendrocyte-specific PERK activation reduced neuron loss in the CNS of EAE mice using the mouse model that allows for temporally controlled activation of PERK specifically in oligodendrocytes. We further generated a mouse model that allows for inactivation of ATF4 specifically in oligodendrocytes, and determined the effects of ATF4 inactivation in oligodendrocytes on mice undergoing EAE.

**Results:**

We showed that protection of oligodendrocytes resulting from PERK activation led to attenuation of neuron loss in the CNS gray matter of EAE mice. Surprisingly, we found that ATF4 inactivation specifically in oligodendrocytes did not alter EAE disease severity and had no effect on oligodendrocyte loss, demyelination, axon degeneration, neuron loss, and inflammation in EAE mice.

**Conclusions:**

These findings suggest the neuroprotective effects of PERK activation in oligodendrocytes in EAE, and rule out the involvement of ATF4 in oligodendrocytes in the development of EAE. These results imply that the protective effects of PERK activation in oligodendrocytes in MS and EAE are not mediated by ATF4.

## Background

Multiple sclerosis (MS) is an autoimmune disease of the central nervous system (CNS) characterized by multi-focal demyelinated plaques in the white matter [1–3]. For many years, the focus of MS research has been on T cell-mediated demyelination of the white matter. Recent studies, however, have shown that neurodegeneration, including axon degeneration and neuron loss, is an early event and the primary cause of chronic disability in MS [4–6]. While oligodendrocytes and myelin are considered to be the primary targets of immune attacks in MS and its animal model experimental autoimmune encephalomyelitis (EAE) [7–9], the mechanisms of neurodegeneration in these diseases remain elusive. Recent studies suggest that oligodendrocytes support axon integrity and neuron survival under normal and diseases conditions [10, 11]. Nevertheless, it remains unclear whether oligodendrocyte death or dysfunction induced by inflammatory attacks contributes to neurodegeneration in MS and EAE.

Pancreatic endoplasmic reticulum kinase (PERK) activation in response to endoplasmic reticulum (ER) stress preserves cell viability and function under stressful conditions by phosphorylating eukaryotic translation initiation factor 2α (eIF2α) [12–14]. Phosphorylated eIF2α (p-eIF2α) inhibits global protein biosynthesis, but stimulates the expression of numerous cytoprotective genes by promoting the translation of activating transcription factor 4 (ATF4). ATF4 also enhances the expression of growth arrest and DNA damage 34 (GADD34), a regulatory subunit of a phosphatase complex that dephosphorylates p-eIF2α, by upregulating CCAAT/enhancer-binding protein homologous protein (CHOP), which forms a negative feedback loop to down-regulate PERK signaling. It has been shown that ER stress is a major player in regulating oligodendrocyte viability in various myelin disorders, including MS and EAE [15–17]. Several studies have demonstrated the cytoprotective effects of the PERK-eIF2α pathway on oligodendrocytes in MS and EAE [18, 19]. Our previous study showed that CNS expression of IFN-γ before EAE onset attenuates the disease severity and ameliorates EAE-induced oligodendrocyte loss, demyelination, and axon degeneration in the CNS and that the beneficial effects of IFN-γ in EAE are associated with activation of the PERK pathway in oligodendrocytes and are abrogated in PERK heterozygous-deficient mice [20]. A report showed that oligodendrocyte-specific PERK inactivation exacerbates EAE disease severity and aggravates EAE-induced oligodendrocyte loss, demyelination, and axon degeneration in the CNS [21]. Moreover, our previous studies showed that activation of PERK specifically in oligodendrocytes protects the cells and myelin against inflammation during EAE [22, 23]. Additionally, a study showed that treatment with Guanabenz, a GADD34 inhibitor, increases the level of p-eIF2α in oligodendrocytes and reduces the disease severity, oligodendrocyte death, and demyelination in EAE mice [24]. Nevertheless, the mechanisms responsible for the cytoprotective effects of the PERK-eIF2α pathway on oligodendrocytes in MS and EAE remain unexplored.

We have generated a mouse model that allows for temporally controlled activation of PERK signaling exclusively in oligodendrocytes and demonstrated that oligodendrocyte-specific PERK activation attenuates oligodendrocyte apoptosis, demyelination, and axon degeneration in the CNS of EAE mice, without altering inflammation [22]. It is believed that inflammation is ultimately responsible for neurodegeneration in MS and EAE [25–27]. This unique mouse model allows us to examine the neuroprotective effects of oligodendrocytes in EAE excluding the influence of inflammation. Herein, we sought to determine whether protection of oligodendrocytes resulting from PERK activation influences neuron viability during EAE. Moreover, we generated a mouse model that allowed for inactivation of ATF4 exclusively in oligodendrocytes and determined the contribution of ATF4 to the protective effects of PERK activation in oligodendrocytes in EAE.

## Methods

### Mice, PCR, and EAE immunization

*PLP*/*Fv2E*-*PERK* mice [22, 23], *ATF4*^*loxP*^ mice [28, 29], and *CNP*/*Cre* mice [30, 31] were on the C57BL/6J background. *PLP*/*Fv2E*-*PERK* mice were maintained by mating with C57BL/6J mice. *ATF4*^*loxP*^ mice were crossed with *CNP*/*Cre* mice, and the resulting offspring were further crossed with *ATF4*^*loxP*^ mice to obtain *ATF4*^*loxP*/*loxP*^; *CNP*/*Cre* mice and *ATF4*^*loxP*/*loxP*^ mice. Genotypes were determined by PCR from DNA extracted from tail tips as described previously [22, 29, 30]. To determine the deletion of exons 2 and 3 of the *Atf4* gene through Cre-Lox recombination in *ATF4*^*loxP*/*loxP*^; *CNP/Cre* mice, genomic DNA was isolated from the indicated tissues and PCR was performed as described in previous papers [28, 29]. To activate Fv2E-PERK in the oligodendrocytes of *PLP*/*Fv2E*-*PERK* mice, the mice were given daily intraperitoneal (i.p.) injections of AP20187 (Ariad Pharmaceuticals, Cambridge, MA) as described in our previous paper [22]; controls were injected with vehicle (4% ethanol, 10% PEG-400, and 2.0% Tween-20 in water) only.

To induce EAE, adult female mice were injected subcutaneously in the flank and at the tail base with 200 μg of myelin oligodendrocyte glycoprotein (MOG) 35–55 peptide emulsified in complete Freund’s adjuvant (BD Biosciences, San Jose, CA, USA) supplemented with 600 μg of *Mycobacterium tuberculosis* (strain H37Ra; BD Biosciences). Two i.p. injections of 400 ng pertussis toxin (List Biological Laboratories, Denver, CO, USA) were given 0 and 48 h later. Clinical scores (0 = healthy, 1 = flaccid tail, 2 = ataxia and/or paresis of hindlimbs, 3 = paralysis of hindlimbs and/or paresis of forelimbs, 4 = tetraparalysis, 5 = moribund or death) were recorded daily as described in our previous papers [22, 31, 32].

All animal procedures were conducted in complete compliance with the National Institutes of Health’s Guide for the Care and Use of Laboratory Animals and were approved by the Institutional Animal Care and Use Committee of the University of Minnesota.

### Western blot analysis

Brains harvested from mice were rinsed in ice-cold PBS and were homogenized using a motorized homogenizer as previously described [31–33]. After incubating on ice for 15 min, the extracts were cleared by centrifugation at 14,000 rpm for 30 min twice. The protein content of each extract was determined by DC Protein Assay (Bio-Rad Laboratories). The extracts (50 μg) were separated by SDS-PAGE and transferred to nitrocellulose. The blots were incubated with a primary antibody against ATF4 (1:4000, Abcam, Cambridge, MA, RRID:AB_940373), CHOP (1:1000, Santa Cruz Biotechnology, Santa Cruz, CA, RRID:AB_783507), or β-actin (1:1000, Sigma-Aldrich, St. Louis, MO, RRID:AB_476694), followed by an HRP-conjugated secondary antibody, and, following incubation with the ECL Detection Reagents (GE Healthcare Biosciences, Pittsburgh, PA), the chemiluminescent signal was detected. The intensity of the recorded chemiluminescence signal was quantified using the ImageQuantTL software from GE Healthcare Life Sciences.

### Immunohistochemistry

Anesthetized mice were perfused through the left cardiac ventricle with 4% paraformaldehyde in PBS. Brains were bisected in the sagittal plane. Both the upper (lumbar 1—lumbar 3) and the lower (lumbar 3–lumbar 5) regions of the lumbar spinal cord were carefully dissected from the vertebra as described in our previous paper [34]. One-half of brains and the spinal cord segments from the lumbar 3 to lumbar 5 were postfixed for at least 48 h in 4% paraformaldehyde in PBS, dehydrated through graded alcohols, and embedded in paraffin. Serial sections of 5 μm thickness were cut. The other half of brains and the spinal cord segments from the lumbar 3 to lumbar 1 were postfixed for 1 h in 4% paraformaldehyde in PBS, cryopreserved in 30% sucrose for 48 h, embedded in OCT compound, and frozen on dry ice. Frozen sections were cut in a cryostat at 10 μm thickness. Immunohistochemistry (IHC) for CC1 (APC7, 1:50; EMD Biosciences, Gibbstown, NJ, RRID:AB_2057371), myelin basic protein (MBP, 1:1000; Sternberger Monoclonals, Berkeley, CA, RRID:AB_10120129), CD3 (1:50; Santa Cruz Biotechnology, RRID:AB_627010), CD11b (1:50; Millipore, Temecula, CA, RRID:AB_92930), NeuN (1:500, Millipore, RRID:AB_2298772), phosphorylated neuro-filament-H (SMI31, 1:1000, Sternberger Monoclonals, RRID:AB_509995), non-phosphorylated neuro-filament-H (SMI32, 1:1000, Covance, San Diego, CA, USA, RRID:AB_509997), CHOP (1:100, Santa Cruz Biotechnology, RRID: AB_783507), calbindin 2 (1:400, Sigma-Aldrich, RRID:AB_476894), and aspartoacylase (ASPA, 1:1000, kindly provided by Dr. M.A. Aryan Namboodiri at Uniformed Services University of the Health Sciences, Bethesda, MD) were performed as described in our previous papers [22, 31, 32, 34]. Signals were detected using Fluorescein (Vector Laboratories, anti-rabbit, RRID:AB_2336197), Cy3 (Millipore, anti-mouse, RRID:AB_11213281, anti-rat, RRID:AB_90854), or enzyme-labeled secondary antibodies (Vector Laboratories, anti-rat, RRID:AB_2336202; anti-mouse, RRID:AB_2313581;, anti-rabbit, RRID:AB_92489). Fluorescent-stained sections were mounted with Vectashield mounting medium with DAPI (Vector Laboratories) and visualized with a Zeiss Axioskop 2 fluorescence microscope (Carl Zeiss Microscopy, Thornwood, NY).

To quantify the cells and axons in the white matter of the lumbar spinal cord, we counted immunopositive cells or axons within the anterior funiculus directly medial to the anterior median fissure in the lumbar spinal cord and confined to an area of 0.1 mm^2^, as described in our previous articles [20, 22, 32, 34]. For quantitative MBP IHC analysis in EAE mice, we calculated the percentage of the demyelinated area in the lumbar spinal cord by normalizing the demyelinated area against the total white matter area. The total white matter area and the demyelinated area in the lumbar spinal cord were measured by NIH ImageJ software (http://rsb.info.nih.gov/ij/; RRID:SCR_003070) as described in our previous paper [20, 22, 32].

To quantify the lower motor neurons in the spinal cord segment from the lumbar 3 to lumbar 5, serial sections of 5 μm thickness were cut and every tenth section was immunostained with the NeuN antibody. The anterior horn of the spinal cord was selected for motor neuron counts. Only cells that had a visible nucleolus, the characteristic morphological features of an α-motor neuron, and a minimum diameter of 13.0 μm were counted using the NIH ImageJ software as described in our previous paper [34]. To quantify the Purkinje neurons in the cerebellum, 5 μm thick sagittal brain sections were cut and every tenth section in the series spanning from Bregma lateral 0.12 mm to 0.36 mm were immunostained with the calbindin 2 antibody. Calbindin 2 positive cells were counted in the lobules I/II, III, and IV of the anterior cerebellum as described in our previous paper [34]. To quantify neurons in the primary motor cortex, 5-μm-thick sagittal brain sections were cut and every tenth section in the series spanning from Bregma lateral 1.08 mm to 1.32 mm were immunostained with the NeuN antibody. NeuN-positive cells were counted in the layer V of the primary motor cortex as described in our previous paper [34].

### T cell proliferation, viability, and cytokine assays

Single-spleen cell suspensions were generated from EAE mice at post-immunization day (PID) 10. The cells were plated in 96-well microtiter plates, treated with MOG35–55 peptide (0, 1, 10, and 100 μg/ml), and incubated at 37 °C and 5% CO_2_ as described in our previous papers [22, 31, 32]. After 48 h, 20 μl of BrdU labeling solution (Millipore) was added to the culture media for 24 h. Cell proliferation was determined using the Colorimetric BrdU Cell Proliferation kit (Millipore) according to the manufacturer’s instructions. We quantified the cytokines in the culture supernatants using the ELISA kits (Thermo Scientific) according to the manufacturer’s instructions. For the T cell viability assay, the spleen cells treated with MOG35–55 peptide were incubated at 37 °C and 5% CO_2_ for 72 h. Cell viability was determined by the MTT assay kit (Progema) according to the manufacturer’s instructions.

### Experimental design and statistics analysis

The sample size for each individual experiment is listed in the corresponding figure legend. For experiments that determine the effects of ATF4 inactivation on oligodendrocytes under normal conditions, both male and female mice were used. For EAE experiments, only female mice were used due to the well-known sex differences in MS. EAE clinical score data are presented as mean ± SEM and are compared using a two-way ANOVA with a Sidak’s multiple-comparisons test using GraphPad Prism 6 Software (RRID:SCR_002798). All other data are presented as mean ± SD. For quantitative analyses, multiple comparisons were statistically evaluated by the one-way ANOVA with Tukey’s posttest using GraphPad Prism 6 Software. Comparison of two groups was statistically evaluated by *t* test using GraphPad Prism 6. *P* < 0.05 was considered significant.

## Results

### PERK activation specifically in oligodendrocytes reduced neuron loss in the CNS of EAE mice

**Fig. 1:**
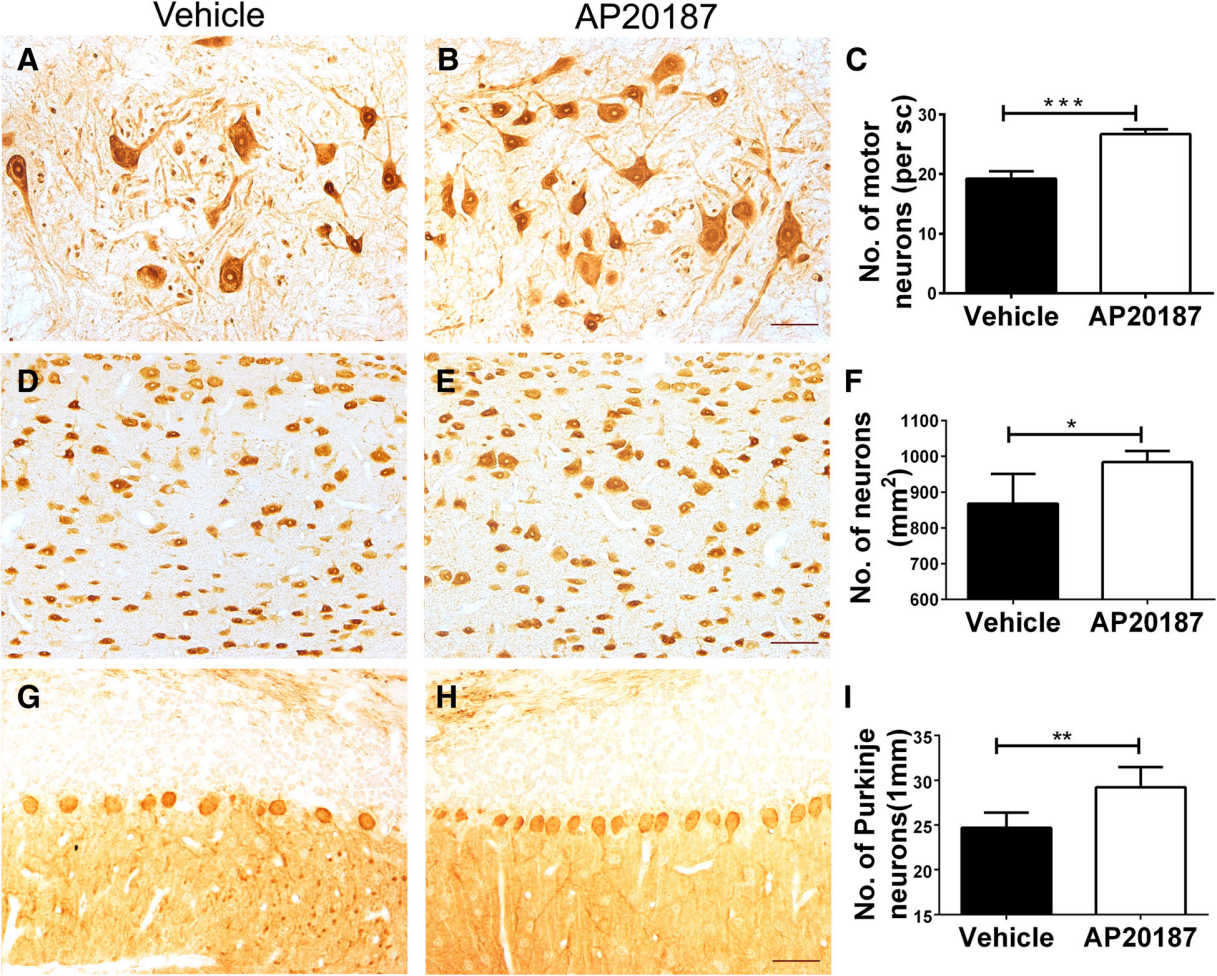
PERK activation specifically in oligodendrocyte attenuated neuron loss in the CNS of EAE mice. **a**–**c** NeuN IHC showed that the number of lower motor neurons was significantly increased in the lumbar spinal cord (SC) of AP20187-treated *PLP*/*Fv2E*-*PERK* mice at PID 21 compared to vehicle-treated mice. **d**– **f** NeuN IHC showed that the number of neurons was significantly increased in the layer V of the primary motor cortex of AP20187-treated *PLP*/*Fv2E*-*PERK* mice at PID 21 compared to vehicle-treated mice. **g**– **i.** Calbindin 2 IHC showed that the number of Purkinje neurons was significantly increased in the cerebellum of AP20187-treated *PLP*/*Fv2E*-*PERK* mice at PID 21 compared to vehicle-treated mice. Scale bars: **a**, **b**, **d**, **e**, **g**, **h**, 50 μm. *N* = 5 animals. Error bars represent SD, *P < 0.05, **P < 0.01, ***P < 0.0001

Seven-week-old female *PLP*/*Fv2E*-*PERK* mice were immunized with MOG35-55 peptide to induce EAE, and then were given i.p. injections of 0.5 mg/kg AP20187 or vehicle daily starting on PID10. Our previous study has demonstrated that AP20187 treatment enhances PERK activation specifically in oligodendrocytes and reduces EAE disease severity and EAE-induced oligodendrocyte apoptosis, demyelination, and axon degeneration, without affecting the inflammatory response [22]. On the other hand, our recent study has demonstrated that the MOG-EAE model displays significant neuron loss in the CNS gray matter, including a significant reduction of lower motor neuron numbers in the lumbar spinal cord, a significant reduction of neuron numbers in the layer V of the primary motor cortex, and a significant reduction of Purkinje neuron numbers in the cerebellum [34]. Herein, we determined the effects of PERK activation in oligodendrocytes on neuron loss in the CNS gray matter during EAE. Quantitative NeuN IHC showed that AP20187 treatment significantly increased lower motor neuron numbers in the lumbar spinal cord (Fig. 1a–c) and neuron numbers in the layer V of the primary motor cortex (Fig. 1d–f) in *PLP*/*Fv2E*-PERK mice at the peak of disease, PID 21. Moreover, quantitative calbindin 2 IHC showed that AP20187 treatment significantly increased Purkinje neuron numbers in the cerebellum of *PLP*/*Fv2E*-*PERK* mice at PID 21 (Fig. 1g–i). Collectively, these data suggest that protection of oligodendrocytes resulting from PERK activation led to attenuation of neuron loss in the CNS gray matter of EAE mice.

### ATF4 was dispensable for oligodendrocytes under normal conditions

ATF4 is the master transcription factor of the PERK-eIF2α pathway, which preserves cell viability and function during ER stress by stimulating the expression of numerous cytoprotective genes [12–14]. It has been demonstrated that neither PERK activation in oligodendrocytes nor PERK inactivation in oligodendrocytes affects the viability or function of oligodendrocytes under normal conditions [21–23]. Nevertheless, global ATF4-deficient mice have a complex phenotype that includes severe anemia, infertility, microphthalmia, growth retardation, and premature death [35, 36]. Therefore, we generated a mouse model that allowed for inactivation of ATF4 specifically in oligodendrocytes and determined the effects of ATF4 inactivation on oligodendrocytes under normal conditions.

**Fig. 2:**
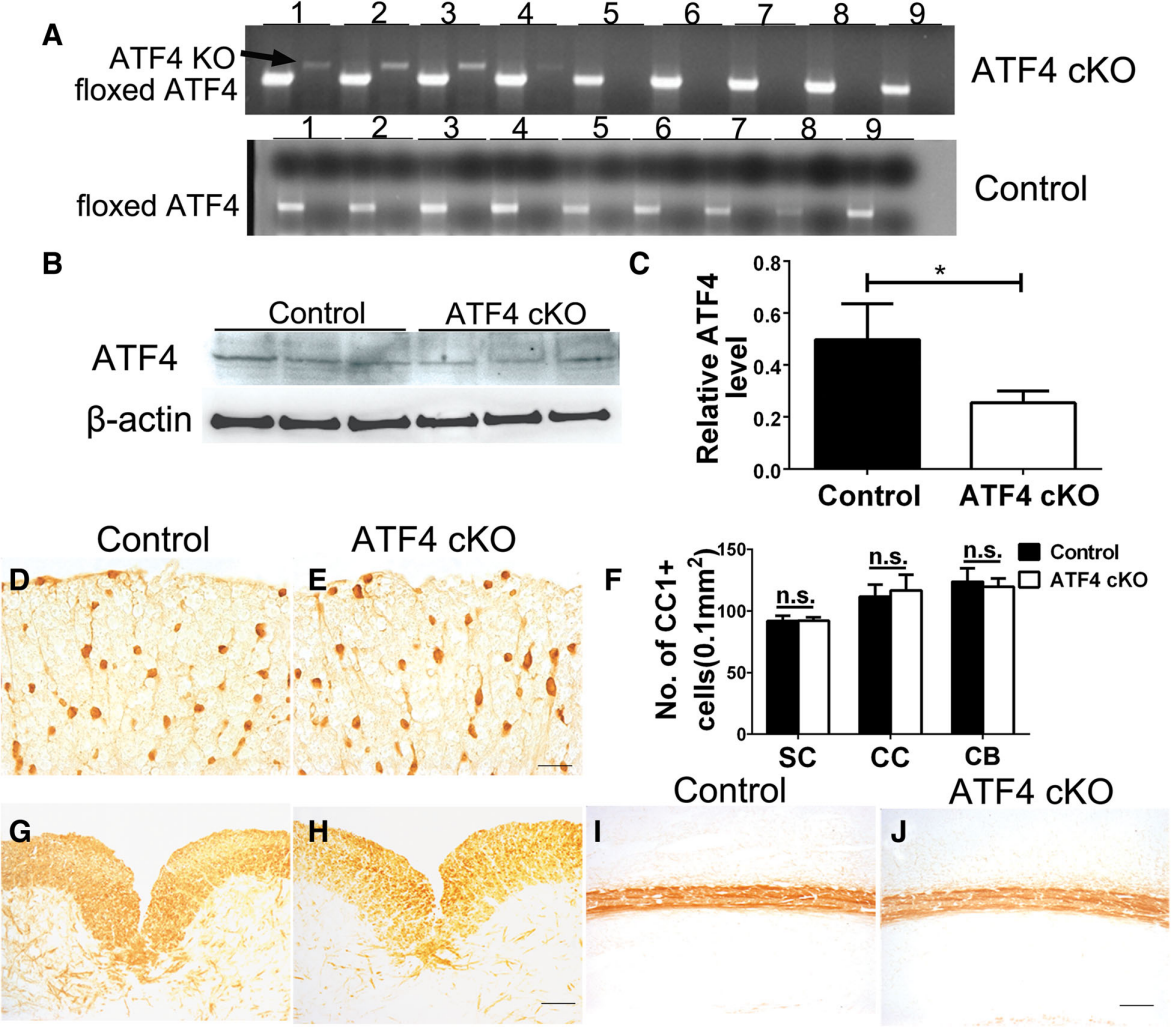
ATF4 inactivation specifically in oligodendrocytes did not affect the viability and function of oligodendrocytes under normal conditions. **a** PCR analysis showed that the floxed ATF4 alleles were presented in the brain (1), spinal cord (2), optic nerve (3), sciatic nerve (4), heart (5), liver (6), spleen (7), lung (8), and kidney (9) of ATF4 cKO mice and control mice; however, ATF4 knockout (KO) alleles were only presented in the brain (1), spinal cord (2), optic nerve (3), and sciatic nerve (4) of ATF4 cKO mice. *N* = 3 animals. **b**, **c** Western blot analysis showed that the protein level of ATF4 was significantly decreased in the brain of 10-week-old ATF4 cKO mice compared to control mice. *N* = 3 animals. **d**–**f** CC1 IHC showed that oligodendrocyte-specific ATF4 inactivation did not significantly change oligodendrocyte numbers in the spinal cord (SC), corpus callosum (CC, representative images not shown), or cerebellum (CB, representative images not shown) of 10-week-old mice. *N* = 4 animals. **g**–**j** MBP IHC showed that oligodendrocyte-specific ATF4 inactivation did not significantly affect the degree of myelination in the spinal cord (**g**, **h**) or corpus callosum (**i**, **j**) of 10-week-old mice. *N* = 4 animals. Scale bars: **d**, **e**, 50 μm; **g**–**j**, 100 μm. Error bars represent SD, *P < 0.05. *n*.*s*. not significant

**Fig. 3:**
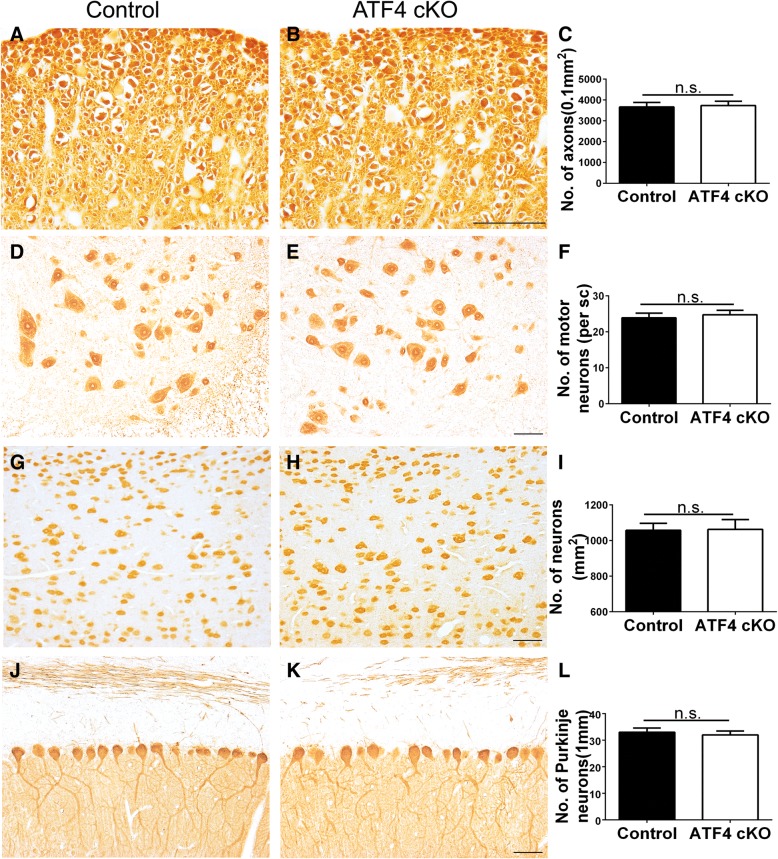
ATF4 inactivation specifically in oligodendrocytes had no effect on axons and neurons in the CNS under normal conditions. **a**–**c** SMI 31 IHC revealed that there was a comparable number of total axons in the lumbar spinal cord of 10-week-old ATF4 cKO mice and control mice. **d**–**f** NeuN IHC showed that ATF4 inactivation specifically in oligodendrocytes did not significantly change the number of lower motor neurons in the lumbar spinal cord of 10-week-old mice. **g**–**i** NeuN IHC showed that ATF4 inactivation in oligodendrocytes did not significantly change the number of neurons in the layer V of the primary motor cortex in 10-week-old mice. **j**–**l** Calbindin 2 IHC showed that there was a comparable number of Purkinje neurons in the cerebellum of 10-week-old ATF4 cKO mice and control mice. Scale bars: **a**, **b**, 20 μm; **d**, **e**, **g**, **h**, **j**, **k**, 50 μm. *N* = 4 animals. Error bars represent SD. *n*.*s*. not significant

*ATF4*^*loxP*^ mice were crossed with *CNP*/*Cre* mice, and the resulting progeny were further crossed with *ATF4*^*loxP*^ mice to obtain *ATF4*^*loxP*/*loxP*^; *CNP*/*Cre* mice (ATF4 cKO mice) and *ATF4*^*loxP*/*loxP*^ mice (control mice). ATF4 cKO mice did not display any neurological phenotypes and were indistinguishable from littermate control mice. PCR analysis confirmed the deletion of exons 2 and 3 of the *Atf4* gene selectively in the CNS and PNS of ATF4 cKO mice, but not in other organs of ATF4 cKO mice or any organs of control mice (Fig. 2a). Western blot analysis revealed the decreased level of ATF4 in the CNS of ATF4 cKO mice compared to control mice (Fig. 2b, c). IHC for CC1, a marker for oligodendrocytes, showed a comparable number of oligodendrocytes in the CNS of 10-week-old ATF4 cKO mice and control mice (Fig. 2d–f). MBP IHC showed that the degree of myelination in the CNS of 10-week-old ATF4 cKO mice was comparable to control mice (Fig. 2g–j). SMI-31 IHC revealed a comparable number of axons in the lumbar spinal cord of 10-week-old ATF4 cKO and control mice (Fig. 3a–c). NeuN IHC showed that ATF4 inactivation in oligodendrocytes did not significantly affect lower motor neuron numbers in the lumbar spinal cord (Fig. 3d–f) or neuron numbers in the layer V of the primary motor cortex (Fig. 3g–i) in 10-week-old mice. Moreover, calbindin 2 IHC showed a comparable number of Purkinje neurons in the cerebellum of 10-week-old ATF4 cKO mice and control mice (Fig. 3j–l). Therefore, these results suggest that ATF4 inactivation specifically in oligodendrocytes has a minimal effect on the CNS under normal conditions.

### ATF4 inactivation specifically in oligodendrocytes did not affect the development of EAE

**Fig. 4:**
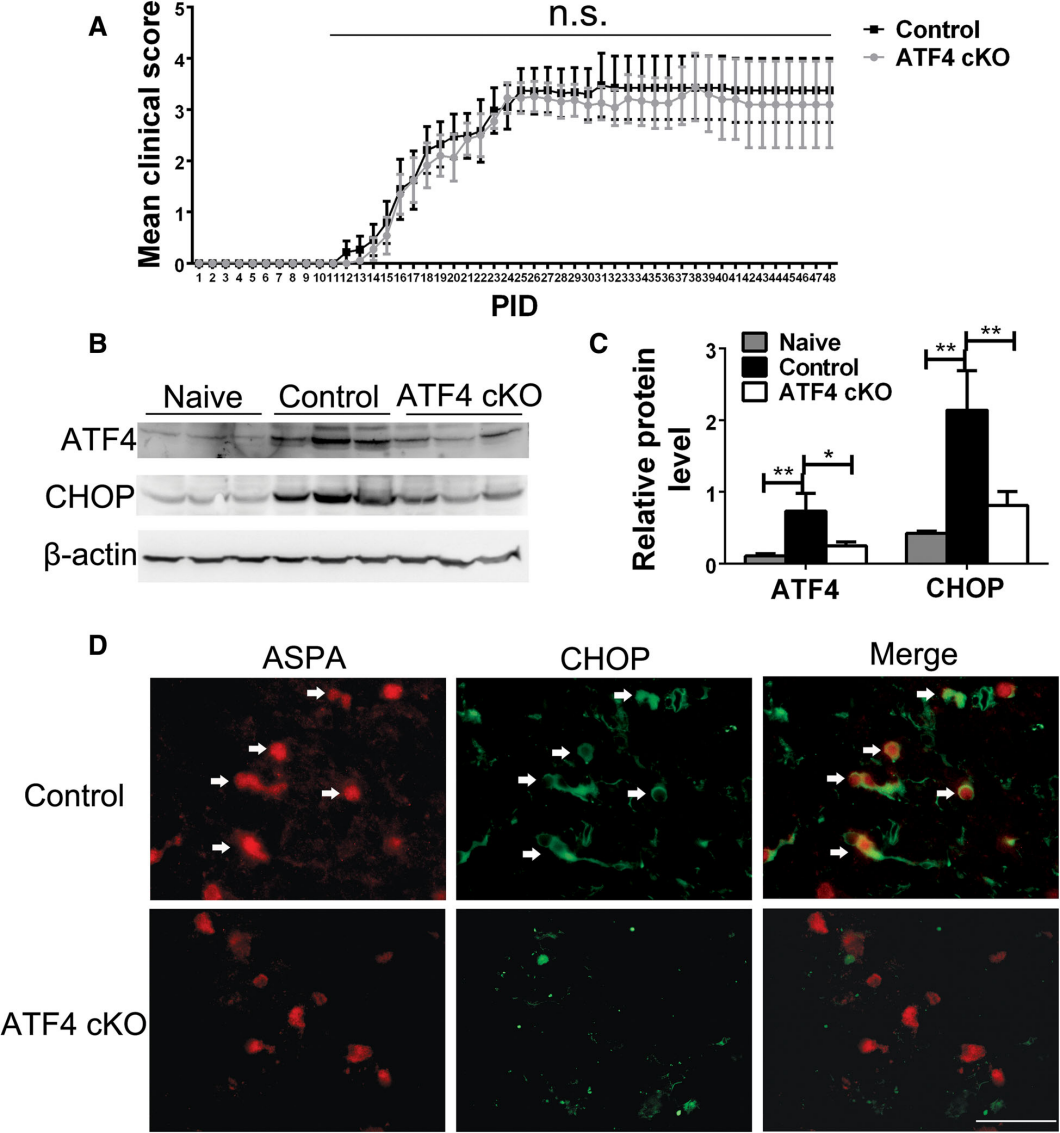
ATF4 inactivation specifically in oligodendrocytes did not alter EAE disease severity. **a** Mean clinical score. Error bars represent SEM, *N* = 9 animals. **b**, **c** Western blots analysis showed that the levels of ATF4 and CHOP were significantly increased in the brain of control mice with EAE at PID 21 compared to naïve control mice. Moreover, the levels of ATF4 and CHOP were significantly decreased in the brain of ATF4 cKO mice with EAE at PID 21 compared to control mice with EAE. *N* = 3 animals. **d** ASPA and CHOP double immunostaining showed that CHOP was detectable in the majority of ASPA positive oligodendrocytes (arrows) in the spinal cord of control mice with EAE at PID21, but was undetectable in ASPA-positive oligodendrocytes of ATF4 cKO mice with EAE. Scale bars: **d**, 20 μm. *N* = 4 animals. Error bars represent SD, *P < 0.05, **P < 0.01. *n*.*s*. not significant

Recent studies have demonstrated the cytoprotective effects of the PERK-eIF2α pathway on oligodendrocytes during EAE [15, 18, 19]. Our previous study demonstrated that PERK activation specifically in oligodendrocytes attenuates axon degeneration during EAE [22]. Herein, we also showed that PERK activation specifically in oligodendrocytes reduced neuron loss during EAE. It is generally believed that the protective effects of the PERK-eIF2α pathway on cells are mediated by its master transcription factor ATF4 [12–14]. Thus, we determined the involvement of ATF4 in the protective effects of PERK activation in oligodendrocytes in EAE using ATF4 cKO mice. Ten-week-old female ATF4 cKO mice and control mice were immunized with MOG35-55 peptide to induce EAE. As expected, control mice exhibited a typical EAE disease course (Fig. 4a). Surprisingly, ATF4 cKO mice displayed a comparable disease course as compared to control mice (Fig. 4a). Western blot analysis showed that the levels of ATF4 and CHOP were significantly increased in the CNS of control mice with EAE at PID21 compared to naïve control mice (Fig. 4b, c). Importantly, the levels of ATF4 and CHOP were significantly decreased in ATF4 cKO mice with EAE at PID21 compared to control mice with EAE (Fig. 4b, c). In accordance with previous studies [20–23], double immunostaining for ASPA, a marker for oligodendrocytes [22, 37], and CHOP showed that the majority of ASPA-positive oligodendrocytes were positive for CHOP in the lumbar spinal cord of control mice with EAE at P21 (Fig. 4d). Nevertheless, none of the ASPA-positive oligodendrocytes were positive for CHOP in the lumbar spinal cord of ATF4 cKO with EAE at P21 (Fig. 4d). These data demonstrate that ATF4 is activated in oligodendrocytes during EAE and that ATF4 activity is abrogated in oligodendrocytes in ATF4 cKO mice with EAE. Collectively, these results suggest that ATF4 activation in oligodendrocytes does not influence EAE disease severity.

**Fig. 5:**
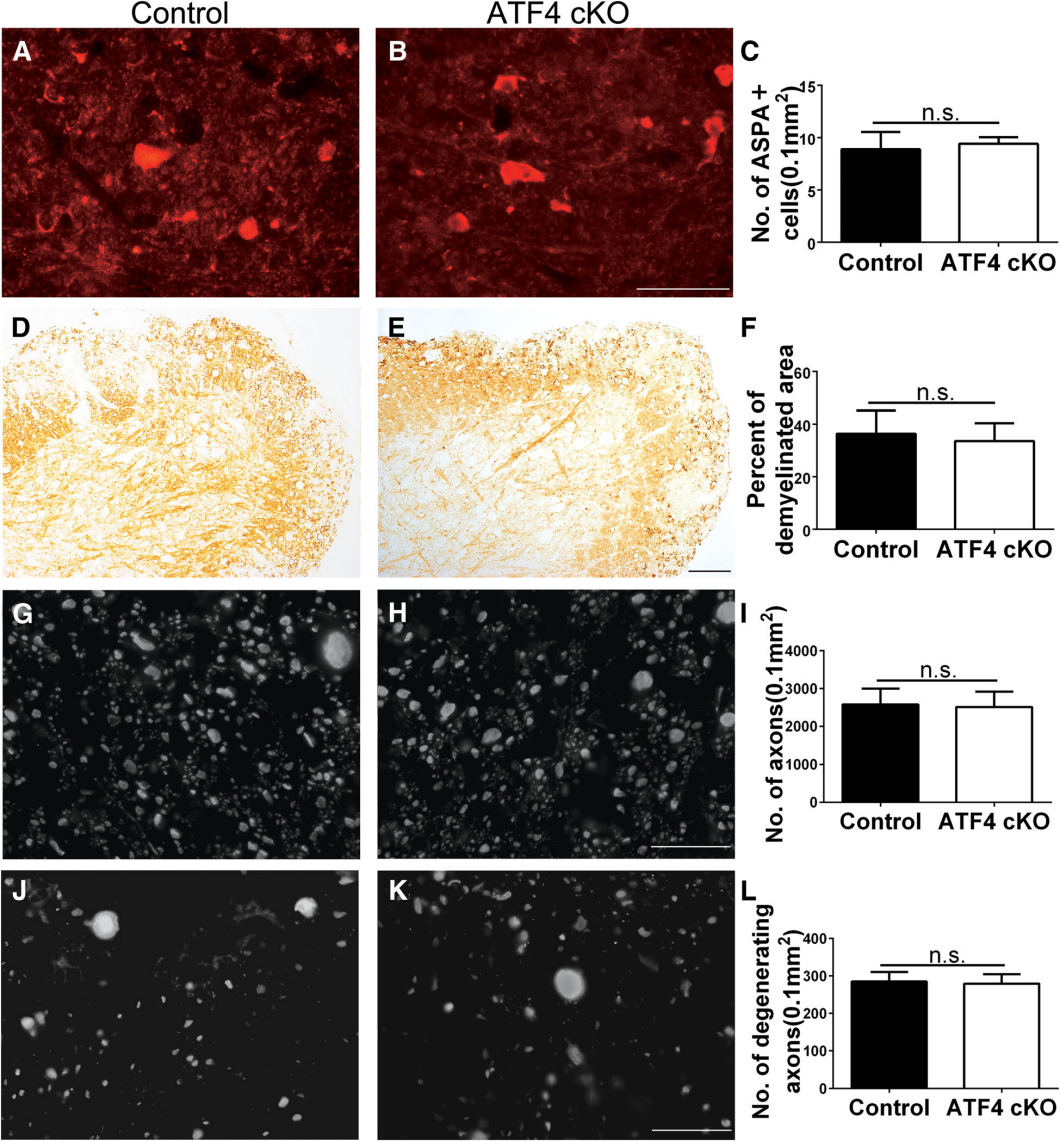
ATF4 inactivation specifically in oligodendrocytes did not alter oligodendrocyte loss, demyelination, or axon degeneration in the CNS of EAE mice. **a**–**c** ASPA immunostaining showed that ATF4 inactivation specifically in oligodendrocytes did not significantly change the degree of reduction of oligodendrocyte numbers in the lumbar spinal cord of EAE mice at PID 21. **d**–**f** MBP IHC showed that ATF4 inactivation specifically in oligodendrocytes did not significantly change the degree of demyelination in the lumbar spinal cord of EAE mice at PID 21. **g**–**i** SMI 31 immunostaining revealed that the number of total axons in the demyelinated lesions in the lumbar spinal cord of ATF4 cKO mice was comparable to control mice at PID21. **j**–**l** SMI 32 immunostaining showed that ATF4 inactivation in oligodendrocytes did not significantly change the number of degenerating axons in the demyelinated lesion in the lumbar spinal cord of EAE mice at PID 21. Scale bars: **a**, **b**, **g**, **h**, **j**, **k**, 20 μm; **d**, **e**, 100 μm; *N* = 4 animals. Error bars represent SD, *n*.*s*. not significant

**Fig. 6:**
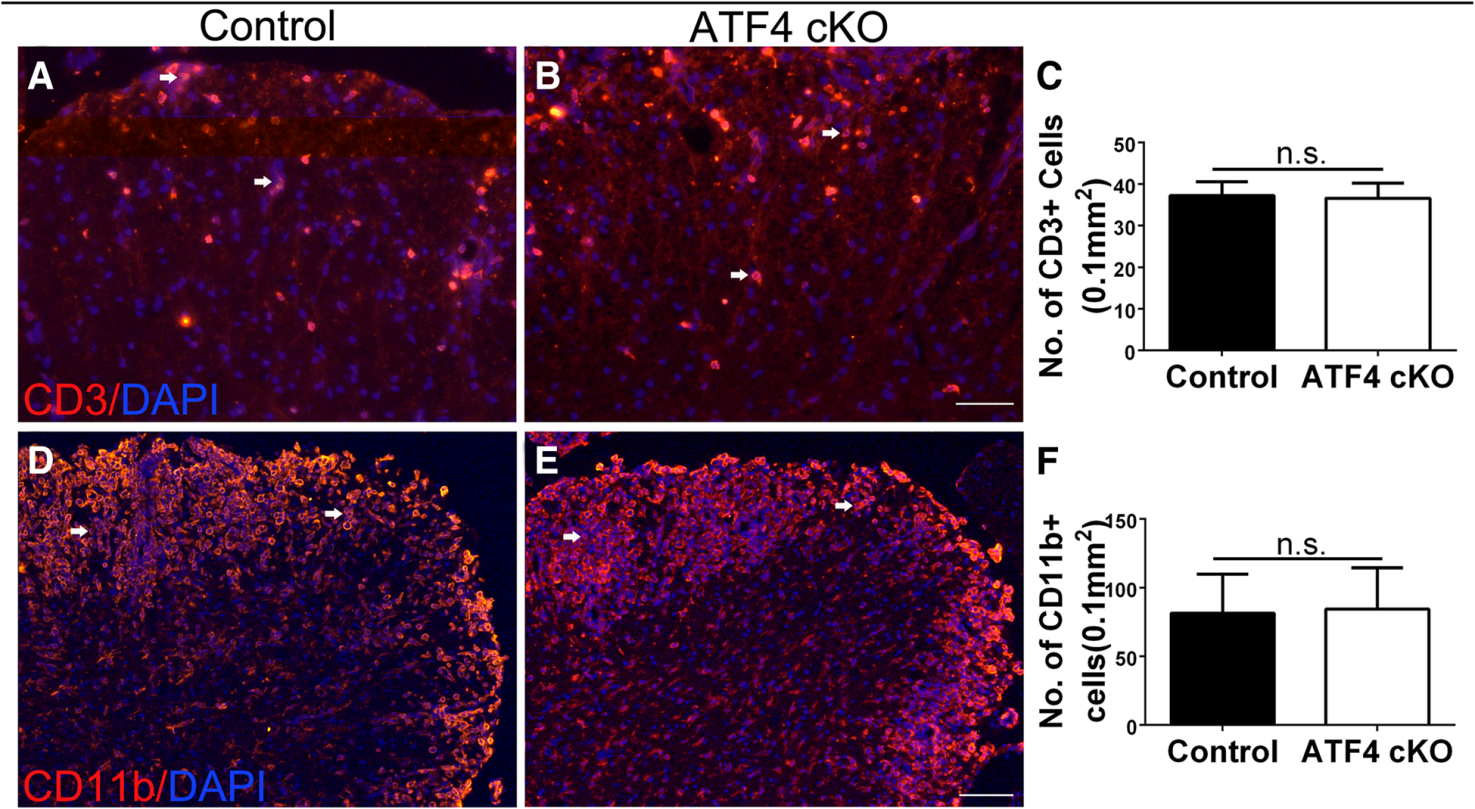
ATF4 inactivation specifically in oligodendrocytes did not change the infiltration of inflammatory cells in the CNS white matter during EAE. **a**–**c** CD3 immunostaining showed that ATF4 inactivation specifically in oligodendrocytes did not significantly change the number of CD3-positive T cells in the white matter of the lumbar spinal cord in EAE mice at PID21. Red fluorescence: CD3, blue fluorescence: DAPI. Arrows pointed out representative CD3-positive T cells. **d**–**f** CD11b immunostaining showed that ATF4 inactivation specifically in oligodendrocytes did not significantly change the number of CD11b positive microglia/macrophages in the white matter of the lumbar spinal cord in EAE mice at PID21. Red fluorescence: CD11b, blue fluorescence: DAPI. Arrows pointed out representative CD11b-positive microglia/macrophages. Scale bars: **a**, **b**, **d**, **e**, 100 μm. *N* = 4 animals. Error bars represent SD, *n*.*s*. not significant

CNS tissues were prepared from these EAE mice at PID21. ASPA immunostaining revealed that there are a few remaining oligodendrocytes in the demyelinated lesions in the lumbar spinal cord of control mice. Unexpectedly, we found that ATF4 inactivation specifically in oligodendrocytes did not significantly change the number of the remaining oligodendrocyte in the demyelinated lesions of EAE mice (Fig. 5a–c). Quantitative analysis of MBP IHC showed that the percentage of area in the white matter of the lumbar spinal cord that was demyelinated in ATF4 cKO mice was comparable with control mice (Fig. 5d–f). Quantitative analysis of SMI-31 immunostaining showed that there was a comparable number of total axons in the demyelinated lesions of ATF4 cKO mice and control mice (Fig. 5g–i). Similarly, quantitative analysis of SMI-32 immunostaining showed that ATF4 inactivation specifically in oligodendrocytes did not significantly alter the number of degenerating axons in the demyelinated lesions of EAE mice (Fig. 5j–l). Moreover, CD3 immunostaining revealed a comparable number of CD3^+^ T cells in the demyelinated lesions in the lumbar spinal cord of ATF4 cKO mice and control mice (Fig. 6a–c). CD11b immunostaining also revealed a comparable number of CD11b^+^ microglia/macrophages in the demyelinated lesions in the lumbar spinal cord of ATF4 cKO mice and control mice (Fig. 6d–f). Taken together, these data suggest that ATF4 activation in oligodendrocytes has no effect on oligodendrocyte loss, demyelination, axon degeneration, and inflammation in the CNS white matter of EAE mice.

**Fig. 7:**
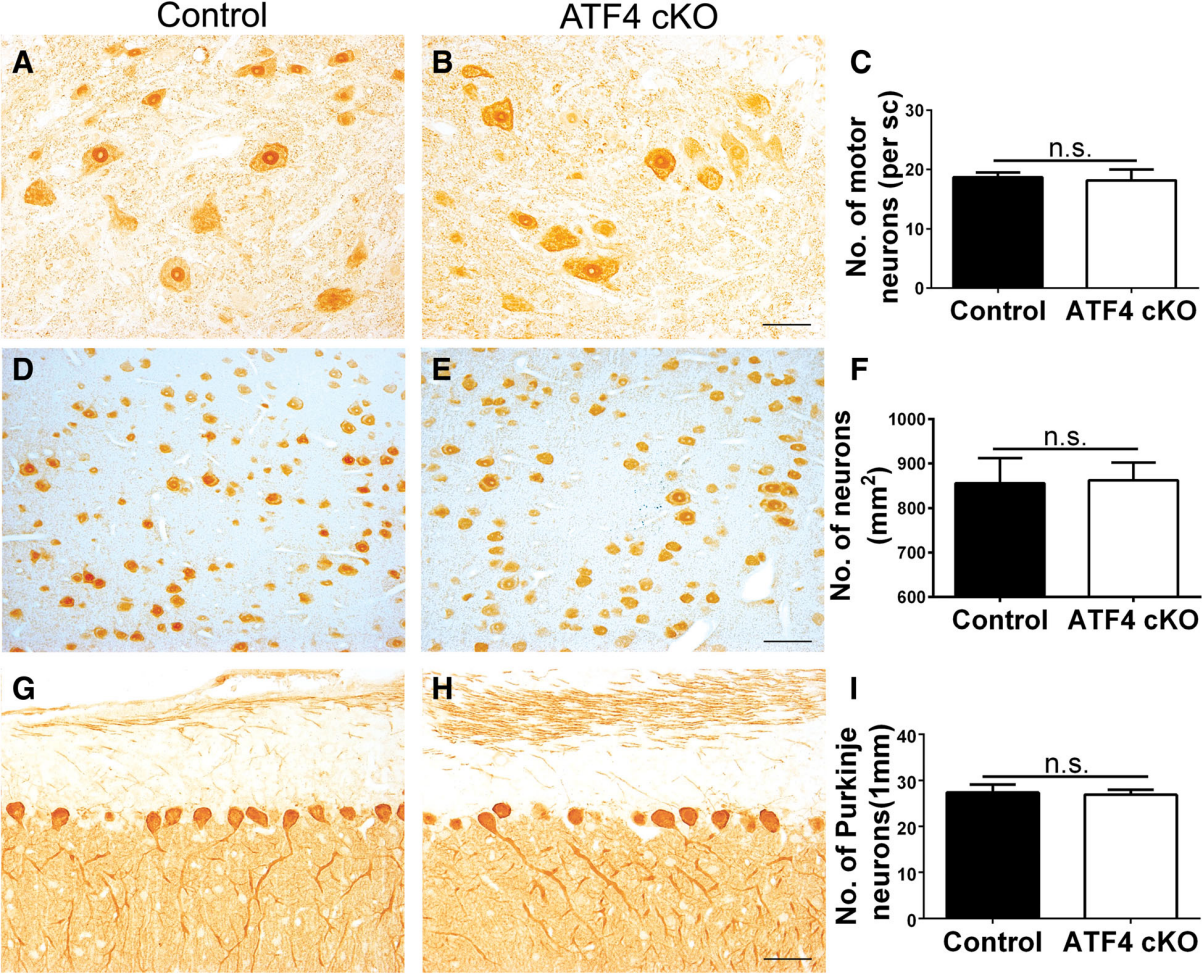
ATF4 inactivation specifically in oligodendrocytes did not affect neuron loss in the CNS gray matter during EAE. **a**–**c** NeuN IHC showed that ATF4 inactivation specifically in oligodendrocytes did not significantly change the number of lower motor neurons in the lumbar spinal cord of EAE mice at PID 21. **d**–**f** NeuN IHC showed that ATF4 inactivation specifically in oligodendrocytes did not significantly change the number of neurons in the layer V of the primary motor cortex in EAE mice at PID 21. **g**–**i** Calbindin 2 IHC showed that ATF4 inactivation specifically in oligodendrocytes did not significantly change the number of Purkinje neurons in the cerebellum of EAE mice at PID 21. Scale bars: **a**, **b**, **d**, **e**, **g**, **h**, 50 μm. *N* = 4 animals. Error bars represent SD, *n*.*s*. not significant

**Fig. 8:**
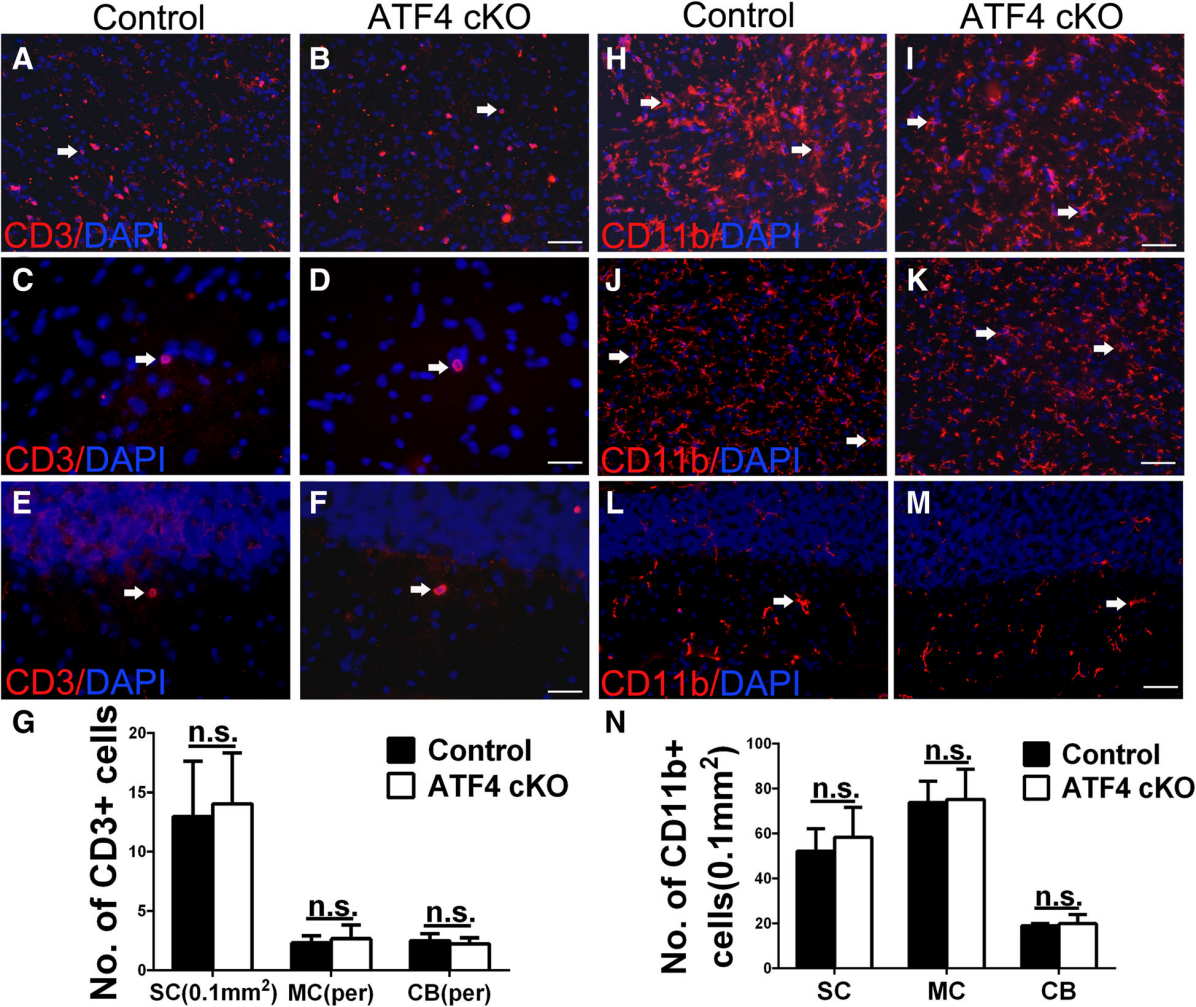
ATF4 inactivation specifically in oligodendrocytes did not change the infiltration of inflammatory cells in the CNS gray matter during EAE**. a**, **b**, **g** CD3 immunostaining showed that ATF4 inactivation specifically in oligodendrocytes did not significantly change the number of CD3-positive T cells in the anterior horn of the spinal cord (SC) in EAE mice at PID21. Red fluorescence: CD3, blue fluorescence: DAPI. Arrows pointed out representative CD3-positive T cells. **c**, **d**, **g** CD3 immunostaining showed that ATF4 inactivation specifically in oligodendrocytes did not significantly change the number of CD3 positive T cells in the layer V of the primary motor cortex (MC) in EAE mice at PID21. Red fluorescence: CD3, blue fluorescence: DAPI. Arrows pointed out representative CD3 positive T cells. **e**–**g** CD3 immunostaining showed that ATF4 inactivation specifically in oligodendrocytes did not significantly change the number of CD3 positive T cells in the molecular layer and internal granular layer of the cerebellum (CB) in EAE mice at PID21. Red fluorescence: CD3, blue fluorescence: DAPI. Arrows pointed out representative CD3-positive T cells. **h**, **i**, **n** CD11b immunostaining showed that ATF4 inactivation specifically in oligodendrocytes did not significantly change the number of CD11b-positive microglia/macrophages in the anterior horn of the spinal cord (SC) in EAE mice at PID21. Red fluorescence: CD11b, blue fluorescence: DAPI. Arrows pointed out representative CD11b-positive microglia/macrophages. **j**, **k**, **n** CD11b immunostaining showed that ATF4 inactivation specifically in oligodendrocytes did not significantly change the number of CD11b-positive microglia/macrophages in the layer V of the primary motor cortex (MC) in EAE mice at PID21. Red fluorescence: CD11b, blue fluorescence: DAPI. Arrows pointed out representative CD11b-positive microglia/macrophages. **l**–**n** CD11b immunostaining showed that ATF4 inactivation specifically in oligodendrocytes did not significantly change the number of CD11b-positive microglia/macrophages in the molecular layer and internal granular layer of the cerebellum (CB) in EAE mice at PID21. Red fluorescence: CD11b, blue fluorescence: DAPI. Arrows pointed out representative CD11b-positive microglia/macrophages. Scale bars: **a–f**, **h**–**m**, 50 μm. *N* = 4 animals. Error bars represent SD, *n*.*s*. not significant

We further determined the potential effects of ATF4 inactivation in oligodendrocytes on neuron loss in CNS gray matter. Quantitative NeuN IHC showed that ATF4 inactivation specifically in oligodendrocytes did not significantly alter the number of lower motor neurons in the lumbar spinal cord (Fig. 7a–c) or the number of neurons in the layer V of the primary motor cortex (Fig. 7d–f) in EAE mice at PID 21. Quantitative calbindin 2 IHC also showed that there was a comparable number of Purkinje neurons in the cerebellum of ATF4 cKO mice and control mice (Fig. 7g–i). Moreover, CD3 immunostaining and CD11b immunostaining revealed comparable T cell infiltration and microglia/macrophage activation in the anterior horn of the spinal cord (Fig. 8a, b, g, h, i, n), the layer V of the primary motor cortex (Fig. 8c, d, g, j, k, n), and the molecular layer and internal granular layer of the cerebellum (Fig. 8e–g, l–n) in ATF4 cKO mice and control mice at PID21. Thus, these data suggest that ATF4 activation in oligodendrocytes has no effect on neuron loss and inflammation in the CNS gray matter of EAE mice.

**Fig. 9:**
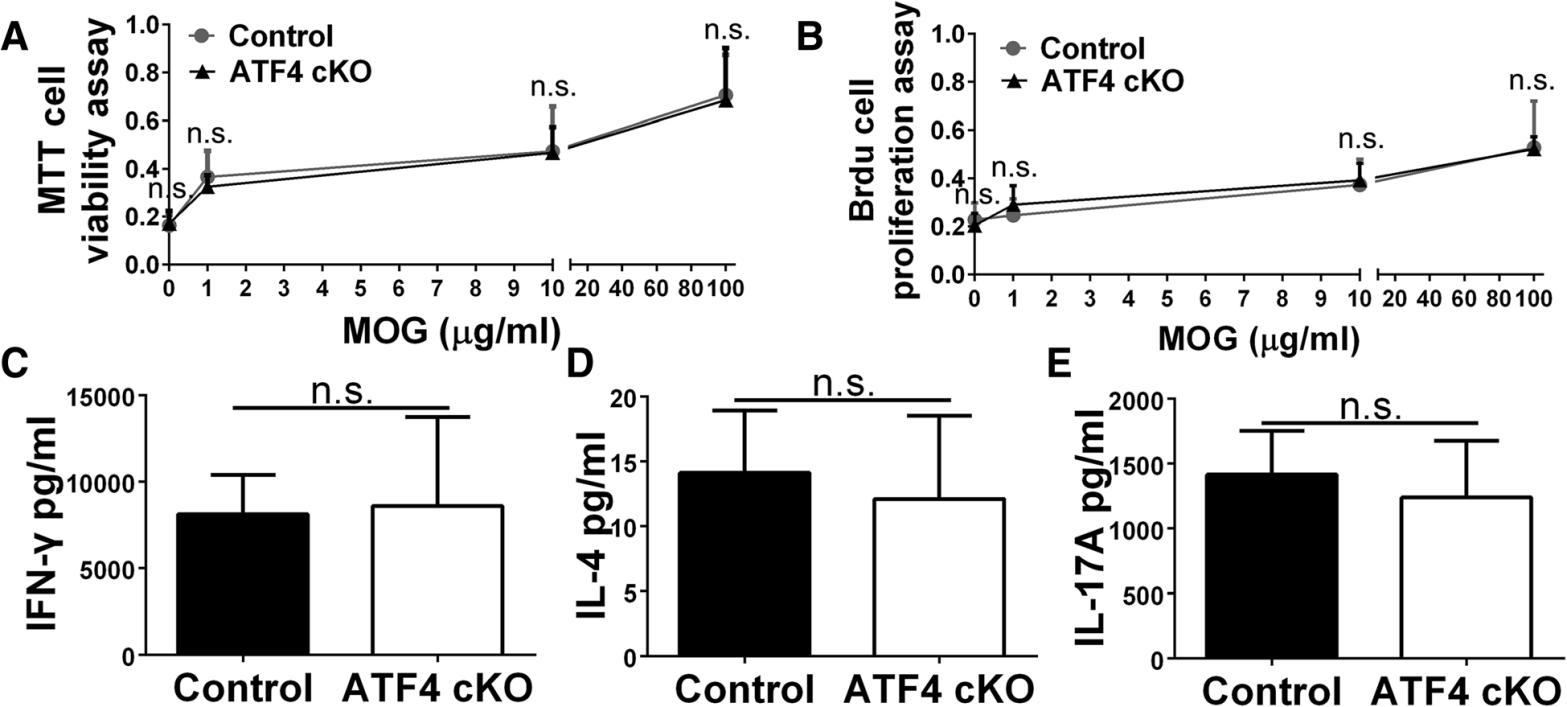
ATF4 inactivation specifically in oligodendrocytes did not affect T cell priming in the peripheral immune system during EAE. **a** The MTT cell viability assay showed that ATF4 inactivation specifically in oligodendrocytes did not significantly alter T cell viability in response to MOG35–55 peptide. **b** BrdU cell proliferation assay showed that ATF4 inactivation specifically in oligodendrocytes did not significantly alter T cell proliferation in response to MOG35–55 peptide. **c**–**e** ELISA analyses showed that ATF4 inactivation specifically in oligodendrocytes did not significantly affect the ability of T cells to produce the cytokines IFN-γ, IL-4, or IL-17A in response to MOG35–55 peptide. *N* = 3 animals. Error bars represent SD. *n*.*s*. not significant

As described above, PCR analysis showed that *Atf4* knockout allele was undetectable in the spleen of ATF4 cKO mice. Next, we performed in vitro T cell recall assays to rule out the possibility that T cell priming was altered in ATF4 cKO mice during EAE. Spleen leukocytes generated from ATF4 cKO mice and control mice at PID10 were re-stimulated with MOG 35-55 peptides. As expected, MTT cell viability assay and BrdU cell proliferation assay showed that the abilities of T cells generated from ATF4 cKO mice to survive and proliferate were not significantly changed compared to those from control mice (Fig. 9a, b). Similarly, ELISA assays showed that the abilities of T cells generated from ATF4 cKO mice to produce the cytokines IFN-γ (Fig. 9c), IL-4 (Fig. 9d), and IL-17A (Fig. 9e) were not significantly changed compared to those from control mice. Thus, these data demonstrate that T cell priming is not altered in ATF4 cKO mice during EAE.

## Discussion

A number of studies have suggested that oligodendrocyte death resulting from immune attacks contributes to the development of MS and EAE [18, 38]. There is evidence that oligodendrocyte apoptosis is the earliest structural change in newly forming demyelinating lesions in MS and EAE [22, 39]. Several reports showed that enhancing oligodendrocyte survival, via enforced expression of anti-apoptotic proteins or deletion of a pro-apoptotic protein in oligodendrocytes, leads to attenuation of oligodendrocyte apoptosis, demyelination, axon degeneration, and inflammation during EAE, and that enhancing oligodendrocyte apoptosis has an opposite effect [40–43]. It is generally believed that inflammation is ultimately responsible for axon degeneration and neuron loss in MS and EAE [25–27]. Because altering oligodendrocyte viability results in a corresponding alteration of inflammation in the mouse models used in the reports described above, these reports cannot differentiate the specific contributions of oligodendrocyte death or inflammation to neurodegeneration during EAE. We have generated a mouse model that allows for temporally controlled activation of PERK exclusively in oligodendrocytes and found that oligodendrocytes-specific PERK activation protects oligodendrocytes against inflammation, but does not alter inflammation in EAE mice [22]. This unique mouse model allows us to dissect the contributions of oligodendrocyte death to neurodegeneration during EAE. Our previous study showed that oligodendrocyte-specific PERK activation attenuates axon degeneration in EAE demyelinated lesions. Herein, we showed that oligodendrocyte-specific PERK activation ameliorated neuron loss in the CNS gray matter during EAE. These results demonstrate that protection of oligodendrocytes resulting from PERK activation prevents axon degeneration and neuron loss in the CNS during EAE. Collectively, these data provide strong evidence that oligodendrocyte death induced by immune attacks contributes to axon degeneration and neuron loss in MS and EAE.

Upon ER stress, induction of ATF4, the master transcription factor of the PERK-eIF2α pathway, preserves cell viability and function in most cell types [12–14]. Data from our lab and other groups have demonstrated the cytoprotective effects of the PERK-eIF2α pathway on oligodendrocytes in animal models of MS [20–24, 33, 44, 45]. In this study, we assessed the possibility that the cytoprotective effects of the PERK-eIF2α pathway on oligodendrocytes in MS and EAE are mediated by ATF4. Although we demonstrated that the activity of ATF4 was significantly enhanced in oligodendrocytes during EAE, it was very surprising to find that ATF4 inactivation specifically in oligodendrocytes did not affect the disease severity or inflammation, and had no effect on oligodendrocyte loss, demyelination, axon degeneration, and neuron loss in EAE mice. Interestingly, in parallel to this finding, a previous report showed that CHOP deficiency does not affect the development of EAE [46]. Thus, these results suggest that ATF4 is not a major player in regulating oligodendrocyte viability during EAE, and rule out the involvement of ATF4 in the cytoprotective effects of the PERK-eIF2α pathway on oligodendrocytes during EAE. On the other hand, it is known that the PERK-eIF2α pathway also activates the transcription factor NF-κB by repressing the translation of the NF-κB inhibitor IκBα [47, 48]. We have demonstrated that PERK activation induces NF-κB activation in oligodendrocytes in in vitro cell cultures and in the EAE model [22, 49]. Importantly, our recent study showed that oligodendrocyte-specific expression of IκBαΔN, a super-suppressor of NF-κB, blocks NF-κB activation selectively in oligodendrocytes and increases the sensitivity of oligodendrocytes to inflammation in animal models of MS [31]. Thus, these data raise the possibility that the cytoprotective effects of the PERK-eIF2α pathway on oligodendrocytes during EAE are mediated by NF-κB.

Oligodendrocytes are responsible for producing myelin that insulates axons [7]. Recent studies also suggest that oligodendrocytes support axon integrity and neuron survival through myelin-independent mechanisms [10, 11]. Deletion of either oligodendrocyte-specific protein CNP or PLP causes widespread axon degeneration in the CNS without apparent myelin abnormalities [30, 50]. Several studies showed that oligodendrocytes promote proximate neuron survival by producing neurotrophic factors, such as brain-derived neurotrophic factor (BDNF) and glial cell-derived neurotrophic factor (GDNF) [51, 52]. Moreover, a number of reports showed that oligodendrocyte-derived lactate is essential for the survival of axons and neurons [53, 54]. We showed here that PERK activation protected oligodendrocytes and myelin against inflammation, and resulted in attenuation of axon degeneration and neuron loss during EAE. It is likely that PERK activation in oligodendrocytes protects axon and neuron against inflammation through both myelin-dependent mechanisms and myelin-independent mechanisms.

A number of studies have shown that activation of the PERK-eIF2α pathway enhances the expression of vascular endothelial growth factor A (VEGF-A), a neurotrophic factor in the CNS [55–57]. VEGF-A promotes axon and neuron survival in various neurodegenerative diseases by binding to VEGF receptor 2 (VEGFR2) [58–60]. Interestingly, our recent study showed that inhibition of VEGFR2 in neurons, via a pharmacological approach, exacerbates axon degeneration and neuron loss during EAE [34]. Moreover, it is known that NF-κB activation can induce VEGF-A expression [61, 62]. Additionally, we found that PERK activation specifically in oligodendrocytes elevated the level of VEGF-A in the CNS of EAE mice (data not shown). Therefore, there is a possibility that PERK activation in oligodendrocytes protects axons and neurons against inflammation during EAE through induction of VEGF-A by activating NF-κB. A mouse model that allows for controllable activation of PERK and inactivation of VEGF-A specifically in oligodendrocytes would be an ideal model to test this possibility.

## Conclusion

In summary, using a mouse model that allows for temporally controlled activation of PERK signaling exclusively in oligodendrocytes, we showed that PERK activation specifically in oligodendrocytes attenuated axon degeneration and neuron loss during EAE. This finding implies the neuroprotective effects of PERK activation in oligodendrocytes in MS and EAE. Moreover, using a mouse model that allows for inactivation of ATF4 exclusively in oligodendrocytes, we showed that ATF4 inactivation specifically in oligodendrocytes had no effect on oligodendrocyte loss, demyelination, axon degeneration, neuron loss, and inflammation in EAE mice. This surprising finding excludes the involvement of ATF4 in the protective effects of PERK activation in oligodendrocytes in MS and EAE. The results presented in this study may lay a foundation for developing therapeutic strategies that prevent not only demyelination but also neurodegeneration in MS patients by targeting the PERK-eIF2α pathway.

## Abbreviations

ASPA: Aspartoacylase
ATF4: Activating transcription factor 4
BDNF: Brain-derived neurotrophic factor
CHOP: CCAAT/enhancer-binding protein homologous protein
CNS: Central nervous system
EAE: Experimental autoimmune encephalomyelitis
eIF2α: Eukaryotic translation initiation factor 2α
ER: Endoplasmic reticulum
GADD34: Growth arrest and DNA damage 34
GDNF: Glial cell-derived neurotrophic factor
IHC: Immunohistochemistry
MBP: Myelin basic protein
MS: Multiple sclerosis
PERK: Pancreatic endoplasmic reticulum kinase
PID: Post-immunization day
SMI31: Phosphorylated neuro-filament-H
SMI32: Non-phosphorylated neuro-filament-H
VEGF-A: Vascular endothelial growth factor A
VEGFR2: VEGF receptor 2
